# Distinct Redox Regulation in Sub-Cellular Compartments in Response to Various Stress Conditions in *Saccharomyces cerevisiae*

**DOI:** 10.1371/journal.pone.0065240

**Published:** 2013-06-07

**Authors:** Anita Ayer, Julia Sanwald, Bethany A. Pillay, Andreas J. Meyer, Gabriel G. Perrone, Ian W. Dawes

**Affiliations:** 1 University of New South Wales, Sydney, Australia; 2 INRES-Chemical Signalling, University of Bonn, Bonn, Germany; 3 University of Western Sydney, Penrith, Australia; Newcastle University, United Kingdom

## Abstract

Responses to many growth and stress conditions are assumed to act via changes to the cellular redox status. However, direct measurement of pH-adjusted redox state during growth and stress has never been carried out. Organellar redox state (*E*_GSH_) was measured using the fluorescent probes roGFP2 and pHluorin in *Saccharomyces cerevisiae*. In particular, we investigated changes in organellar redox state in response to various growth and stress conditions to better understand the relationship between redox-, oxidative- and environmental stress response systems. *E*_GSH_ values of the cytosol, mitochondrial matrix and peroxisome were determined in exponential and stationary phase in various media. These values (−340 to −350 mV) were more reducing than previously reported. Interestingly, sub-cellular redox state remained unchanged when cells were challenged with stresses previously reported to affect redox homeostasis. Only hydrogen peroxide and heat stress significantly altered organellar redox state. Hydrogen peroxide stress altered the redox state of the glutathione disulfide/glutathione couple (GSSG, 2H^+^/2GSH) and pH. Recovery from moderate hydrogen peroxide stress was most rapid in the cytosol, followed by the mitochondrial matrix, with the peroxisome the least able to recover. Conversely, the bulk of the redox shift observed during heat stress resulted from alterations in pH and not the GSSG, 2H^+^/2GSH couple. This study presents the first direct measurement of pH-adjusted redox state in sub-cellular compartments during growth and stress conditions. Redox state is distinctly regulated in organelles and data presented challenge the notion that perturbation of redox state is central in the response to many stress conditions.

## Introduction

Eukaryotic organisms have evolved to maintain distinct redox environments in organelles, allowing compartmental specific processes to occur. A highly reducing environment is maintained in compartments including the cytosol, mitochondrial matrix and peroxisome to facilitate proper protein folding and activity. Conversely, the endoplasmic reticulum and mitochondrial intermembrane space have been observed to maintain a more oxidizing environment [1], [2]. Aberrant sub-cellular redox environments can be detrimental, affecting a broad range of cellular processes including signal transduction, RNA, DNA and protein synthesis [3]–[5] and cell cycle regulation [6], [7]. As a result, maintaining appropriate redox homeostasis is critical for cellular function [8]. Redox dysfunction has been implicated in various stress conditions including osmotic, heat and oxidative stress through protein and microarray data, and resistance/sensitivity phenotypes [9]–[13]. However the exact change to sub-cellular redox state under various stress conditions is unknown.

Cellular redox homeostasis can be significantly affected in cells via mechanisms that alter the state of key redox couples and systems such as the glutathione disulfide/glutathione (GSSG/2GSH) and NADP^+^/NADPH couples. Additionally, alterations to cellular pH can significantly affect redox homeostasis since many redox reactions are pH dependent [8]. For example, the reduction potential of the GSSG/2GSH couple changes by 59 mV between pH 7 and 8 which alters the effectiveness of glutathione as a redox buffer.

Conventionally, the pH and redox environment of cells have been measured using *in vitro* assays or dyes. Such methods prohibit the measurement of redox and pH state in an accurate and real-time manner in sub-cellular compartments. The spatiotemporal pattern of intracellular redox and pH changes may differ between cellular compartments especially after stress, and understanding the significance of redox and pH state changes at a compartmental level is extremely important to an understanding of the regulation of these two processes. Development of redox- and pH-responsive fluorescent probes such as roGFP2 [14] and pHluorin [15] have allowed dynamic *in vivo* analysis of redox state and pH. The sensitivity of these fluorescent proteins was achieved by mutagenesis of wild type green fluorescent protein (GFP). In these GFP variants, changes to redox state or pH alter the structure of GFP leading to alterations in fluorescence emission after excitation at two different wavelengths. By measuring the changes to emission after excitation at 405 nm and 488 nm in cells expressing roGFP2 or pHluorin the redox state and pH of an intracellular compartment can be calculated accurately. As the roGFP2 probes equilibrates predominantly with the GSSG/2GSH couple and is stable between pH 5–8 [16], analyses using targeted roGFP2 and pHluorin probes allow reliable estimation of the sub-cellular state of the glutathione disulfide/glutathione couple adjusted for pH. Unlike in animal cells, roGFP2 in *S. cerevisiae* responds dynamically to physiological changes in the GSSG/2GSH couple. Consequently, by using organelle-targeted roGFP2/pHluorin constructs in tandem, the contribution of organelles to the overall cellular redox environment under various stress and physiological states can be defined.

Here, we investigated the response of the GSSG/2GSH redox state in *Saccharomyces cerevisiae* to conditions that have previously been linked to redox dysfunction. Specifically, these analyses were carried out on a compartmental level, investigating the cytosol, mitochondrial matrix and peroxisome using roGFP2 and pHluorin constructs genetically targeted at these organelles. Organellar redox state was determined under various physiological states, with redox state significantly affected by growth phase and carbon source. The sub-cellular redox state was also determined when cells were challenged with common environmental stresses. Both hydrogen peroxide and heat shock significantly altered sub-cellular redox state via two different mechanisms while osmotic stress and exposure to superoxide generators did not alter redox state, highlighting the need to understand the relationship between stress conditions and sub-cellular redox state.

## Materials and Methods

### Yeast Strains and Growth Medium

*S. cerevisiae* strains of the BY4743 background were used for all analyses (EUROSCARF). Cells were either grown in fermentative synthetic complete medium lacking uracil (SC_URA_; 2% w/v D-glucose, 0.17% yeast nitrogen base lacking ammonium and amino acids (BD Difco, Sydney, NSW) and 0.5% ammonium sulphate) or respiratory synthetic complete medium lacking uracil (SGE_URA_; 2% w/v glycerol, 1% ethanol, 0.17% yeast nitrogen base lacking ammonium sulphate and amino acids (BD Difco, Sydney, NSW) and 0.5% ammonium sulphate). Both media were supplemented as listed in Table S1. All analyses were carried out in SC_URA_ in exponential phase unless otherwise stated. Exponential phase refers to cell cultures with an A_600_ = 0.5–1 and stationary phase refers to cell cultures with an A_600_ = 5–6. For continuous monitoring of cell growth cultures were incubated with shaking at 30°C in a Bioscreen C (Oy Growth Curves AB Ltd).

### Plasmids

Details of organelle-targeted roGFP2 constructs pAG416-roGFP2 and pAG416-COX4-roGFP2 are described in [17] and pAG416-roGFP2SKL [18]. For details of roGFP2-SKL localization analyses and calibration see [18].

Organelle-targeted pHluorin probes were constructed using PCR amplification and the Invitrogen Gateway System® (Invitrogen Life Technologies, Carlsbad, CA) in a manner analogous to the construction of the organelle-targeted roGFP2 robes. Briefly, the pH-sensitive green fluorescent protein (pHluorin) open reading frame was amplified using PCR from pCB901YpHc (a kind gift from R. Rao). For pAG416-COX4-pHluorin, pHluorin was PCR amplified and the matrix targeting sequence (codons for amino acids 1–25 of the cytochrome oxidase subunit IV gene COX4) was fused at the *N*-terminus of pHluorin. For the peroxisomal-targeted pHluorin (pAG416-pHluorin-SKL), the peroxisomal *C-*terminal targeting sequence encoding seryl-lysyl-leucine was added on to the primer used for amplification. *attb1* and *attb2* sites were added to primers as appropriate so that the resulting localization signal containing pHluorin sequence was flanked by a 5′ *attb1* site and a 3′ *attb2* site. The resulting PCR fragments were used for the Gateway BP® clonase® reaction using the destination vector pDonr221 generating the plasmids pDonr221-COX4-pHluorin and pDonr221-pHluorin-SKL. These plasmids were used for the Gateway LR® clonase® reaction using the destination vector pAG416GPD-ccdB [19]. All reactions were carried out according to manufacturer’s instructions. Primers used are given in Table S2.

### Localization of pHluorin and Organelle-targeted Derivatives

Correct localization of organelle-targeted pHluorin was confirmed by confocal microscopy. Localization of the pHluorin probes was determined using confocal microscopy. For localization analyses cells were grown and treated as follows: 1) cells expressing cytosolically localized pHluorin were grown in SC_URA_ overnight and analyzed microscopically; 2) cells expressing the matrix-targeted pHluorin (COX4-pHluorin) were grown in SC_URA_ overnight and incubated with DAPI (Sigma Aldrich, St Louis, MO) for 30 min to stain DNA; 3) cells expressing the peroxisomal-targeted pHluorin were grown in SC_URA_ overnight and analyzed microscopically. Cells were examined using an Olympus FV-1000 confocal microscope at a magnification of 100× and Brightfield and fluorescence images. Brightfield, GFP and DAPI were visualized using the 543-, 488-, and 405 nm lasers respectively.

### Analysis of Compartmental Redox State (E_GSH_)

BY4743 cells transformed with the roGFP2 constructs were grown in SC_URA_ for 2 d (30°C; 700 rpm), inoculated in SC_URA_ medium and grown with shaking at 25°C until appropriate cell yield had been reached. For analysis of cells in respiratory medium, cells transformed with the roGFP2 constructs were grown in SC_URA_ for 2 d (30°C; 700 rpm), inoculated in SGE_URA_ medium and grown with shaking at 25°C until appropriate cell yield had been reached. Cells were treated as outlined below and then analyzed using the FACS Canto II (BD Bioscience) instrument equipped with a laser that excites at 405 nm and 488 nm. Emission was detected at 512 nm. The ratio of the emission after excitation at 405 nm to that at 488 nm was then calculated. Cells were also treated for 20 min with 10 mM DTT (Astral Scientific, Sydney, NSW) or 10 mM diamide (Sigma Aldrich, St Louis, MO) to obtain measurements of the fully reduced or oxidized (respectively) forms of the roGFP2 probe that are necessary to calculate *E*_GSH_ as previously reported [20]. *E*_GSH_ was then calculated as previously described [17] with 10,000 cells counted for each sample. The pH value obtained using pHluorin was used for calculation of *E*_GSH_. Data obtained were analyzed with FlowJo version 7.4 software.

### Analysis of Compartmental pH

BY4743 cells transformed with the relevant pHluorin construct were grown in SC_URA_ for 2 d (30°C; 700 rpm), inoculated in SC_URA_ medium and grown with shaking at 25°C until appropriate cell yield had been reached. For analysis of cells in respiratory medium, cells transformed with the pHluorin construct were grown in SC_URA_ for 2 d (30°C; 700 rpm), inoculated in SGE_URA_ medium and grown with shaking at 25°C until appropriate cell yield had been reached. Cells were treated as outlined below and then analyzed using the FACS Canto II (BD Bioscience) as described above. To obtain pH values using the pHluorin probe in each compartment, a standard curve was established to allow estimation of compartmental pH by determining the fluorescence of digitonin-permeabilized cells incubated in buffers with pH values of 5 to 8 [21]–[23]. For calibration of the cytosolically-, mitochondrial matrix-, and peroxisomal-localized pHluorin, cells were suspended in 0.65 M sorbitol containing 100 mg/ml digitonin (Sigma Aldrich, St. Louis, MO) and incubated for 10 min with shaking (600 rpm; 24°C). Cells were pelleted, washed and resuspended in calibration buffer containing 50 mM 2-(*N*-morpholino)ethanesulfonic acid (MES; Sigma Aldrich, St. Louis, MO), 50 mM *N*-(2-hydroxyethyl)piperazine-Ń-(2-ethanesulfonic acid) (HEPES; Sigma Aldrich, St. Louis, MO), 50 M KCl (Ajax Lab Chemical, Sydney, NSW), 50 mM NaCl (Ajax Lab Chemical, Sydney, NSW), 0.2 M ammonium acetate (Ajax Lab Chemical, Sydney, NSW), 10 mM NaN_3_ (Ajax Lab Chemical, Sydney, NSW), 10 mM 2-deoxyglucose (Sigma Aldrich, St. Louis, MO), 75 µM monensin (Sigma Aldrich, St. Louis, MO) and 10 µM nigericin (Sigma Aldrich, St. Louis, MO), titrated to different pH values (6.0–8.0) using NaOH in 0.5 pH unit increments. Calibration curves were plotted using the ratio of fluorescence intensity at 405 nm and 488 nm (after background subtraction) against pH. 10,000 cells were counted for each sample and data obtained were analyzed with FlowJo version 7.4 software.

### ß -galactoside Assay

The cellular heat shock response was measured using the HSE2-lacZ and HSE12-lacZ heat shock reporter constructs [24]. The HSE2-LacZ construct contains an *E. coli lacZ* gene encoding ß-galactosidase under the control of the heat shock factor binding motif and the HSE12-lacZ construct contains an *E. coli lacZ* gene encoding ß-galactosidase linked to a scrambled, heat -inactive promoter. Cells transformed with these constructs were grown to exponential phase (A_600_ = ∼0.5), harvested and analyzed for beta -galactosidase activity [25]. ß-galactosidase activity was expressed as units of ONPG (*O*-nitrophenyl-ß-D-galactopyranoside) hydrolyzed (nmol) per minute over total protein (mg). For each condition, three independent transformants were assayed.

### Propidium Iodide Staining

Cells were harvested by centrifugation, resuspended in 1 ml phosphate buffered saline (PBS) and stained with propidium iodide (10 µg/ml) for 20 min in the dark, washed twice with PBS, and the level of PI staining analysed by microscopy and flow cytometry. Stained cells were analysed with either a FACS Canto II (BD Bioscience) instrument equipped with a laser that excites at 488 nm and emission was detected at 617 nm using a 556 nm long pass filter an a 585/42 broad pass filter or a Leica fluorescence microscope.

### Statistical Analyses

For analysis of significance of cell yield and *E*GSH data, unpaired t-tests were conducted on samples with *p* = 0.01. For data discussed in the text, if a significant difference was found, this is indicated by the inclusion of a *p*-value.

## Results

**Figure 1:**
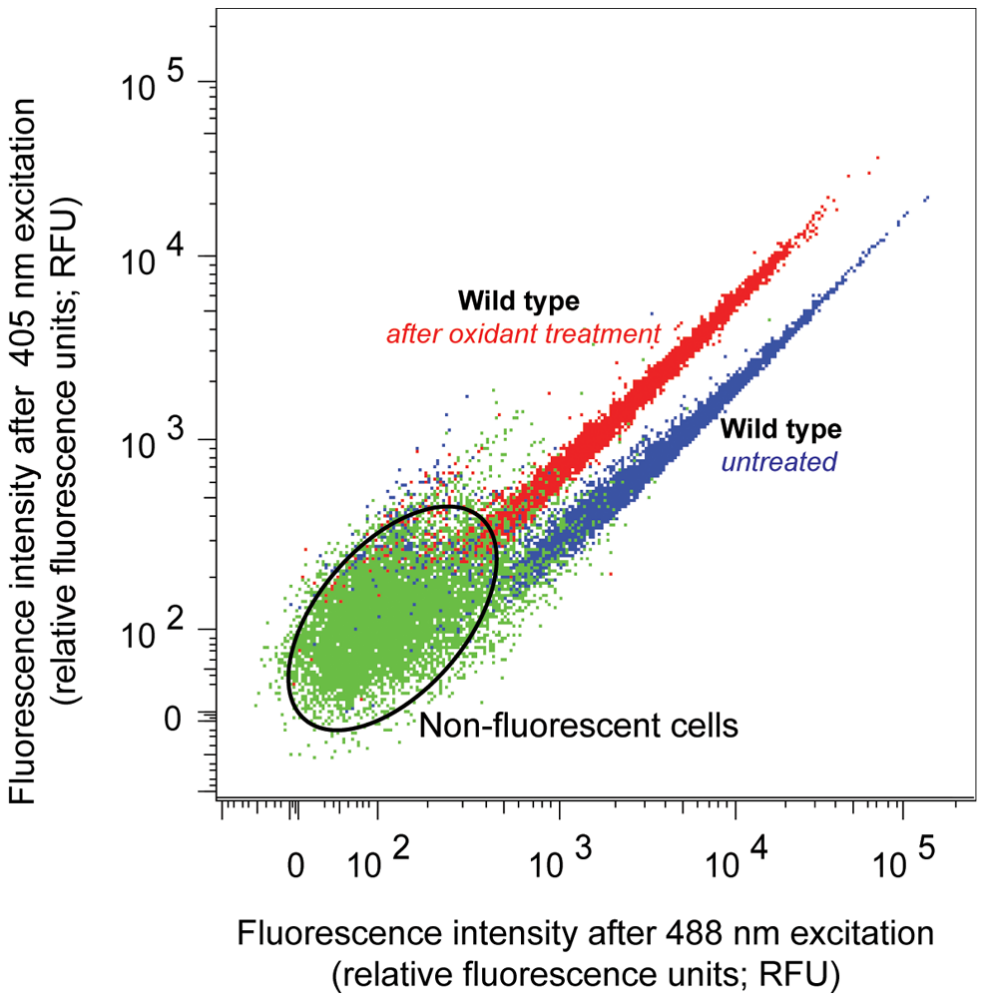
Representative dot plot of cells expressing cytosolic roGFP2. Cells expressing cytosolic roGFP2 were grown to exponential phase (A_600_∼0.5) in SC_URA_ and analyzed by flow cytometry. Dot plot of wild-type cells untreated and treated with oxidant (4 mM hydrogen peroxide). Note the shift in the populations after treatment with hydrogen peroxide towards 405 nm. Dot plots were generated using FlowJo™ software.

All analyses using roGFP2 and pHluorin were performed using flow cytometry (FACS). For FACS analyses, the mean of the population was used to determine redox state and pH after background fluorescence was subtracted with 10,000 cells counted, with non-fluorescent cells making up less than 20% of the population. A representative dot plot generated from flow cytometry analysis of cells expressing cytosolic roGFP2 is shown in Figure 1. Redox and pH shifts were indicated by an increase in emission (after excitation at 405 nm and a simultaneous decrease in emission after excitation at 488 nm) in the test population relative to the control population. Populations generally had a small standard deviation indicating that redox state and pH were maintained across a narrow range in most cells or growth condition. Thus, the redox potentials and pH values obtained reflected the majority of the cell populations studied.

**Figure 2:**
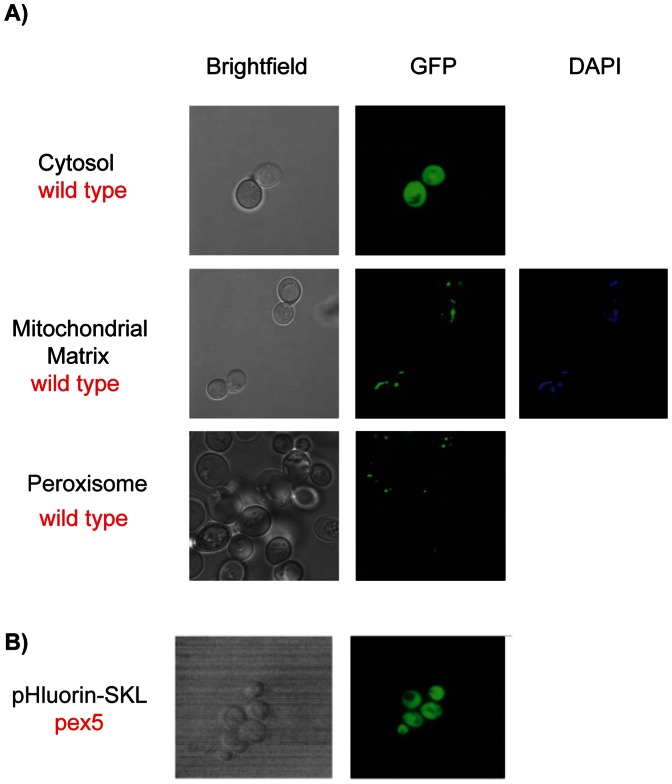
Confocal microscopy images of yeast cells expressing cytosolic, mitochondrial matrix or peroxisomal pHluorin. A) Wild type cells were pregrown in SC_URA_ (48 h; 30°C; 600 rpm), inoculated into SC_URA_ (A_600_ = 0.001), grown until exponential phase and examined using an Olympus FV-1000 confocal microscope at a magnification of 100X. B) In cells where mitochondrial DNA was visualized cells were incubated with DAPI for 30 min, washed with sterile water and examined. C) *pex5* cells were pregrown in SC_URA_ (48 h; 30°C; 600 rpm), inoculated into SC_URA_ (A_600_ = 0.001), grown to exponential phase (A_600_ = ∼0.5) and examined using an Olympus V-1000 confocal microscope at a magnification of 100X.

The roGFP2 probes targeted to the cytosol and mitochondrial matrix and peroxisome were generated and verified as described previously [17], [18]. Localization of organelle targeted pHluorin constructs was analyzed by confocal microscopy (Figure 2A–B) using established markers. Appropriate localization of pHluorin probes was determined in an analogous manner to roGFP2 analyses. To verify specific localization of mitochondrial matrix-targeted pHluorin to mitochondria, cells were grown overnight in SC_URA_ and stained with the mitochondrial marker 4′,6-diamidino-2-phenylindole (DAPI). Mitochondrial matrix-targeted pHluorin localized with DAPI indicating specific mitochondrial matrix localization (Figure 2A). Specific localization of the pHluorin-SKL construct to the peroxisome was demonstrated using the same technique of Van Roermund et al [23] and Ayer et al [18]. In wild-type cells expressing the pHluorin-SKL construct, a punctate fluorescent pattern consistent with peroxisomal localization was observed. We then expressed pHluorin-SKL in *pex5* cells, which are defective in the import of peroxisomal proteins with a peroxisomal targeting signal 1 (PTS1) such as -SKL. In *pex5* cells expressing pHluorin-SKL, the punctate fluorescent pattern seen in wild-type cells was abolished (Figure 2B). Instead a cytosolic fluorescent pattern was observed indicating that in the wild type the SKL-tagged constructs were correctly targeted and are imported via the PTS1-mediated import pathway into the peroxisome.

**Figure 3:**
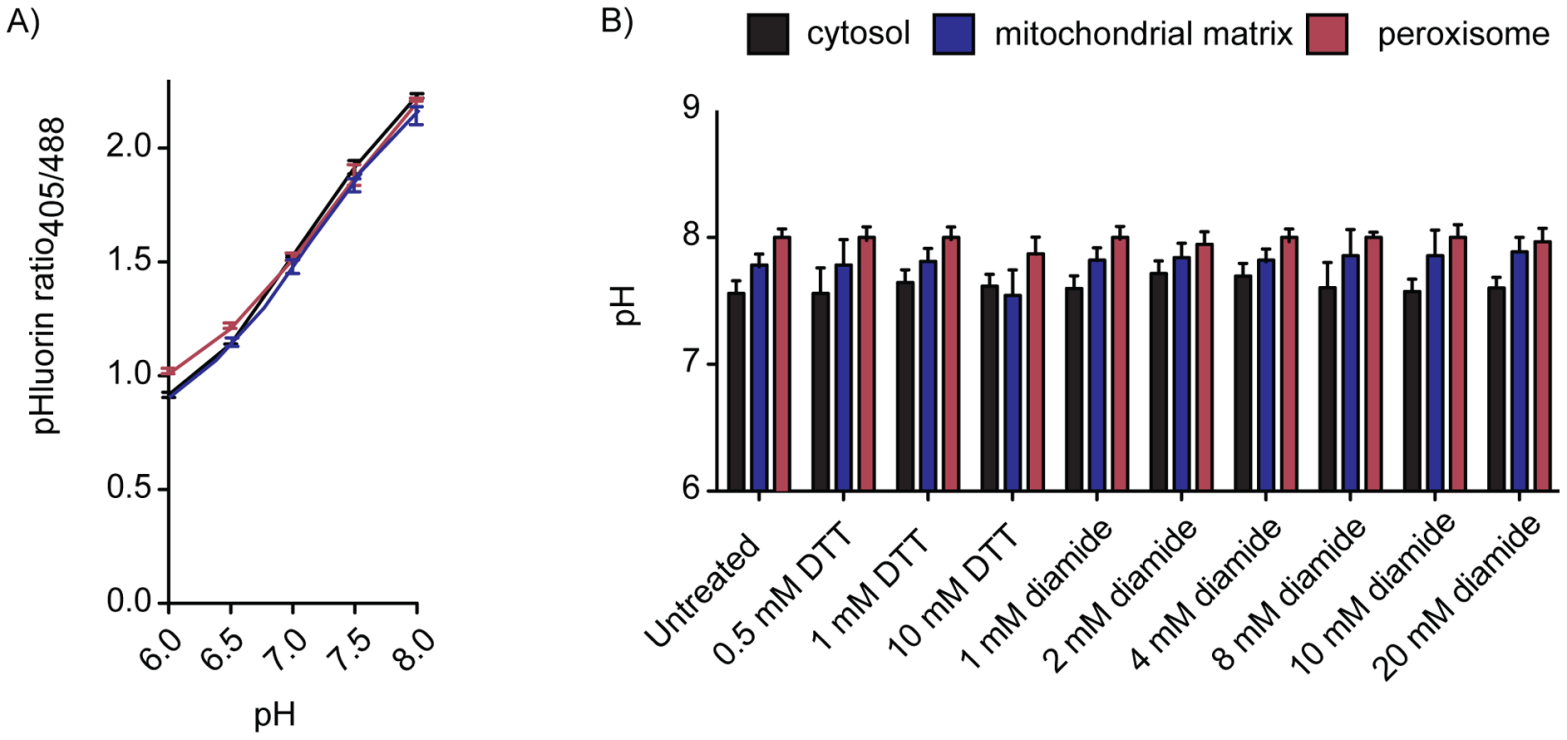
Verification of organelle-targeted pHluorin responsiveness to pH changes. A) pHluorin calibration curves: Cells expressing compartment targeted-pHluorin were pregrown in SC_URA_ (48 h; 30°C; 600 rpm), inoculated in SC_URA_ (A_600_ = 0.001) and grown (25°C; 600 rpm) until exponential phase (A_600_ = ∼0.5). Cells were treated with digitonin (100 µg/ml; 10 min; 600 rpm), resuspended in a sorbitol/digitonin solution and then incubated with calibration buffer adjusted to various pH. Cells were analyzed via flow cytometry with 10,000 cells counted for each sample. Calibration curves are indicated for the cytosol (black line), mitochondrial matrix (grey line) and peroxisome (dashed line). B) Effect of diamide treatment on pH as measured by pHluorin in wild-type cells: cells expressing the compartment-targeted pHluorin probes were pregrown in SC_URA_ (48 h; 30°C; 600 rpm), inoculated in SC_URA_ (A_600_ = 0.001) and grown (25°C; 600 rpm) until exponential phase (A_600_ = ∼0.5). Cells were treated with diamide or DTT (30 min; 600 rpm), and analyzed via flow cytometry.

The response of roGFP2 probes to redox state has been calibrated *in vitro*, its stability over an intracellular pH range of 5–8 has also been established [14], [16] and the responsiveness to *in vivo* redox state change has been confirmed [18]. pHluorin sensitivity to pH was also verified by the construction of calibration curves after incubation of digitonin-permeabilized cells for 50 minutes in various buffers between pH 6.0–8.0. Calibration curves were plotted using the ratio of fluorescence intensity at 405 nm and 488 nm (after background subtraction) against pH for each targeted construct (Figure 3A). All constructs responded to virtually the same extent after digitonin-treatment and incubation in various pH solutions indicating that pHluorin responded equally in all compartments tested. In each respective condition tested, pH was measured using pHluorin and used to calculate *E*_GSH_. To verify that changes in pHluorin were redox-independent, pH was determined in wild-type cells transformed with pHluorin constructs treated with DTT, a strong thiol reductant, or diamide, a strong oxidizer of glutathione. No significant change in pH was observed in any compartment upon treatment with DTT or diamide, indicating that changes to pHluorin are specific to changes in intracellular pH and that the pHluorin probes are redox-independent (Figure 3B).

### Redox State in Sub-cellular Compartments is Differentially Affected by Growth Phase and Carbon Source

It is known that changes in carbon source lead to alterations in macromolecule composition and fluxes of redox intermediates through central metabolic pathways [26]. We therefore decided to examine the effect of carbon source and growth phase on sub-cellular redox state. To investigate if the redox state of the cytosol, mitochondrial matrix and peroxisome were affected by growth phase or carbon source, *E*_GSH_ and pH values were determined in cells transformed with the appropriate constructs during the two main phases of growth (exponential and stationary phase) in both fermentative (SC_URA_; glucose) and respiratory (SGE_URA_; glycerol/ethanol) medium. For cells grown fermentatively, the redox states of the cytosol, mitochondrial matrix and peroxisome were estimated to be highly reducing, with the *E*_GSH_ between −340 to −350 mV (Table 1).

**Table 1:** *E*_GSH_ and pH of wild type cells grown in fermentative (SD) and respiratory (SGE) medium in exponential and stationary phase.

	*E*_GSH_ ± SD (−mV)			pH ± SD		
	Cytosol	Matrix	Peroxisome	Cytosol	Matrix	Peroxisome
**Condition**						
SD; exponential phase	350±3	357±3	342±4	7.5±0.2	7.7±0.2	8.0±0.2
SD; stationary phase	335±3	348±3	340±4	7.5±0.2	7.7±0.1	8.0±0.1
SGE; exponential phase	342±5	339±4	340±3	7.5±0.1	7.7±0.2	8.0±0.2
SGE; stationary phase	335±4	338±3	341±4	7.1±0.2	7.3±0.1	7.6±0.1

Peroxisomal *E*_GSH_ was not affected by growth phase or carbon source. However, cytosolic and mitochondrial matrix *E*_GSH_ were. With respect to growth phase, both cytosolic and mitochondrial matrix *E*_GSH_ became more oxidized by 10–15 mV in stationary phase relative to exponential phase in cells grown in glucose (fermentative conditions). This may be expected since cells grown in glucose will have shifted from fermentation to respiration during the diauxic shift prior to stationary phase. It is likely that under respiratory conditions the *E*_GSH_ will be more oxidizing, due to increased function of the respiratory chain. This was confirmed since the cytosol and matrix redox state were more oxidizing in exponential phase in cells grown on respiratory medium (SGE; glycerol/ethanol) compared to cells growing on fermentative medium (SD; glucose). Moreover, there was no significant shift in redox state between exponential and stationary-phase cells grown in glycerol/ethanol. These results show that the major physiological component in setting *E*_GSH_ is whether the cells are respiring or not, not the phase of growth. Most clearly, the cytosol and mitochondrial matrix in cells grown under respiratory conditions (stationary phase or growth during any phase in glycerol/ethanol) showed a 10–15 mV shift to more oxidizing conditions compared to fermentative growth (exponential phase in glucose) indicating the effect of respiration on sub-cellular redox state.

The *E*_GSH_ values presented are more reducing than previously estimated [2], [27]–[29]. This resulted from the measured pH values of the cytosol and mitochondrial matrix (pH 7.5 and 7.7 respectively) being more basic than those previously estimated [30]. When wild-type cells were grown to stationary phase in respiratory medium, the pH of the cytosol, mitochondrial matrix and peroxisome decreased by approximately 0.4 pH units compared to the pH of cells grown in fermentative medium indicating a change in both redox state and pH as cells switched to respiration.

### Sub-cellular Responses to Hydrogen Peroxide and Paraquat

The transcriptional, proteomic and sensitivity/resistance phenotypes of cells treated with H_2_O_2_ and paraquat (which leads to generation in cells of the superoxide anion radical; O_2_^.-^) have been extensively studied [9], [31]–[33] with the functions involved in the responses to, and tolerance of, H_2_O_2_ and O_2_^.-^ well documented. While both agents can be deleterious for growth and cell function, the manner in which these two reactive oxygen species (ROS) generators affect the sub-cellular redox environment is less well understood. Dardalhon et al [34] have studied the effects of H_2_O_2_ treatment on cytoplasmic and nuclear redox state using the redox sensitive probe rxYFP. However there has been no direct study of the effects of H_2_O_2_ on the redox state of the peroxisome or mitochondrial matrix or of superoxide generators in any compartment.

To test the effects of paraquat on the sub-cellular redox state, cells expressing organelle-targeted roGFP2 and pHluorin were grown to exponential phase, treated with 2 mM paraquat for one hour and analyzed immediately for *E*_GSH_ and pH. Despite this dose of paraquat leading to increased transcription of Mn superoxide dismutase gene [35], there was no significant change of *E*_GSH_ or pH in the cytosol, mitochondrial matrix or peroxisome (Table 2). From these data, it is unlikely that paraquat or the resulting superoxide anion radical formation affects pH or the redox state of the glutathione redox couple in wild-type cells. This is supported by fact that deletion of *SOD1*, the cytosolic superoxide dismutase had no effect on cytosolic or mitochondrial *E*_GSH_ [18].

**Table 2:** *E*_GSH_ and pH of wild type cells grown in SC_URA_ to exponential phase and treated with various stressors for 1 hour.

	*E*_GSH_ ± SD (-mV)			pH ± SD		
	Cytosol	Matrix	Peroxisome	Cytosol	Matrix	Peroxisome
**Condition**						
Untreated	350±3	357±3	346±3	7.5±0.1	7.7±0.2	8.0±0.2
Paraquat (2 mM)	352±4	353±3	347±2	7.5±0.2	7.7±0.2	8.0±0.1
Sorbitol (1.8 M)	350±2	355±4	345±3	7.5±0.1	7.7±0.1	8.0±0.1
KCl (1.3 M)	345±5	357±4	345±3	7.5±0.2	7.7±0.2	8.0±0.2
NaCl (0.9 m)	344±4	354±5	342±6	7.5±0.1	7.7±0.1	8.0±0.2

**Figure 4:**
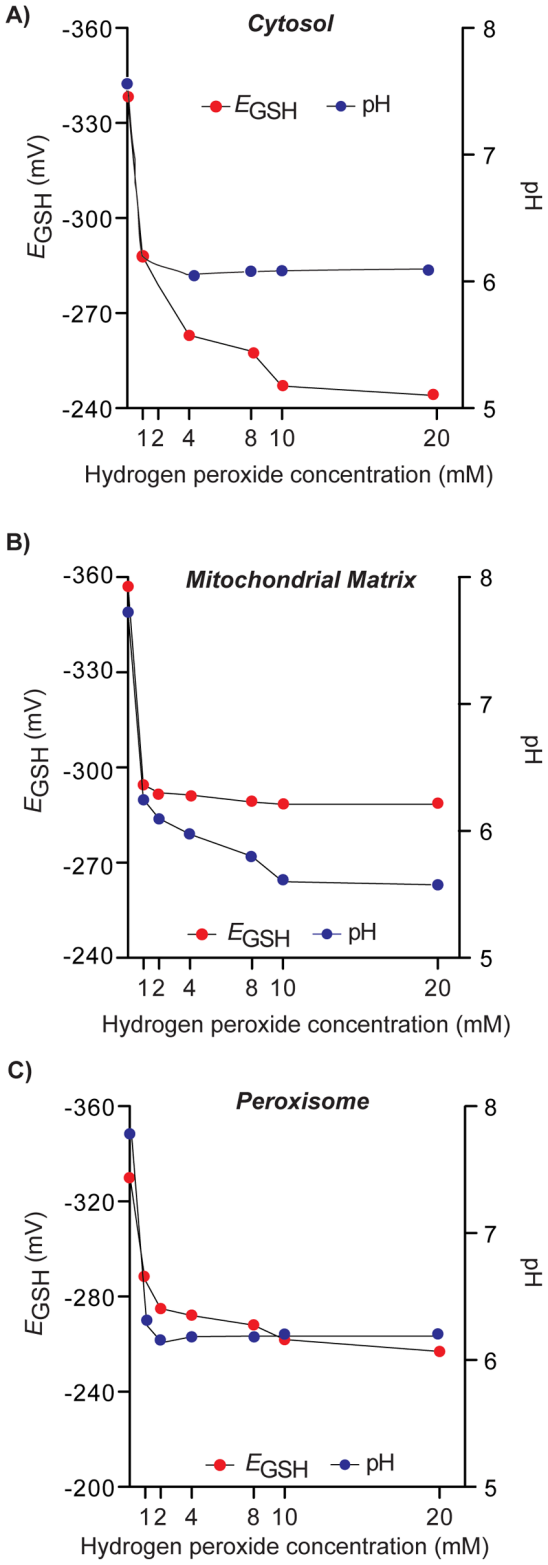
Effect of hydrogen peroxide on compartmental *E*_GSH_ and pH. A-C) Cells were pregrown in SC_URA_ (48 h; 30°C; 600 rpm), inoculated in SC_URA_ (A_600_ = 0.001) and grown (25°C; 600 rpm) until exponential phase (A_600_ = ∼0.5). Cells were then treated with various doses of hydrogen peroxide for 30 min and pH and redox environment analyzed. (A cytosolic pH and *E*_GSH_, B) mitochondrial matrix pH and *E*_GSH_, C) peroxisomal pH and *E*_GSH_. 10,000 cells were counted for each sample. Each experiment was conducted in triplicate with error bars representing the standard deviation of three experiments. Error bars may be difficult to visualise as the errors are in the ∼5 mV range.

To test how rapidly *E*_GSH_ changes occurred following hydrogen peroxide treatment, cells treated with 2 mM hydrogen peroxide were analysed at five-minute intervals for 30 minutes. *E*_GSH_ changes were observed as early as 5 minutes post hydrogen peroxide addition, and this change was maximal as no further *E*_GSH_ changes were observed during the 30 minute treatment. To further investigate the effects of hydrogen peroxide on sub-cellular *E*_GSH_, wild-type cells expressing roGFP2 or pHluorin were treated with various concentration of H_2_O_2_ (1–20 mM) for 30 min and redox state and pH analyzed in the cytosol, mitochondrial matrix and peroxisome (Figure 4A–C). All compartments tested followed a similar trend in *E*_GSH_ upon hydrogen peroxide treatment. The redox state of every compartment tested became more oxidized with increasing doses of hydrogen peroxide. As little as 1 mM H_2_O_2_ led to a significant shift of *E*_GSH_ in all compartments tested (*p<*0.01), with a Δ*E*_GSH_ of 40–50 mV after treatment. Treatment with 20 mM hydrogen peroxide shifted the roGFP2 probe near its oxidized limit with a Δ*E*_GSH_ of ∼100 mV after treatment with 20 mM H_2_O_2_. While this is a high concentration of H_2_O_2_, it does indicate the sensitivity and range of roGFP2 *in vivo*. Interestingly, treatment with H_2_O_2_ affected the pH of each compartment significantly (*p*<0.01). The pH of the cytosol, mitochondrial matrix and peroxisome all decreased by approximately 1 pH unit after 30 minutes of H_2_O_2_ treatment and this pH shift contributed significantly to the observed changes in redox state changes.

**Figure 5:**
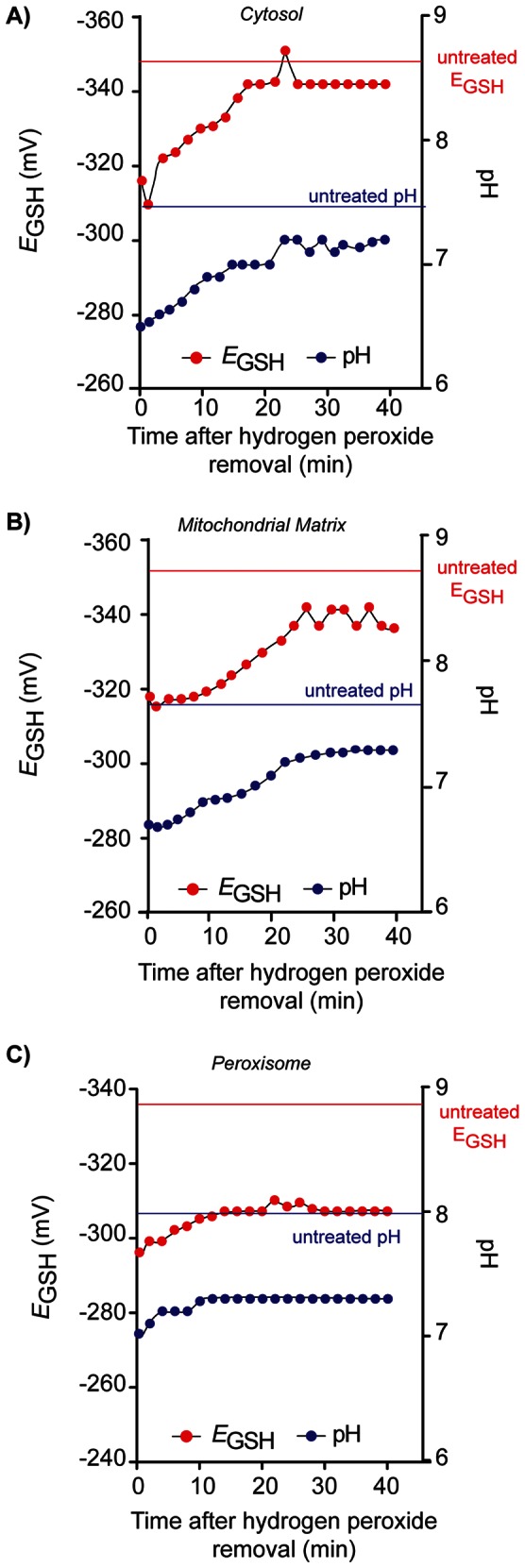
Dynamic recovery of cells after hydrogen peroxide treatment. Cells were pregrown in SC_URA_ (48 h; 30°C; 600 rpm), inoculated in SC_URA_ (A_600_ = 0.001) and grown (25°C; 600 rpm) until exponential phase (A_600_ = ∼0.5). Starting *E*_GSH_ was measured then cells were treated with hydrogen peroxide (2 mM; 20 min; 25°C), washed and resuspended in fresh medium, and pH and redox state analyzed by flow cytometry at two-minute intervals. A) cytosolic pH and *E*_GSH_, B) mitochondrial matrix pH and *E*_GSH_ and C) peroxisomal pH and *E*_GSH._ 10,000 cells were counted for each sample. Each experiment was conducted in triplicate with error bars representing the standard deviation of three experiments. The E_GSH_ and pH in untreated (control cells) is indicated by the marked dashed lines. Some error bars may be difficult to visualise as the errors are in the ∼5 mV range.

An important aspect in the physiology of cells under stress is the manner in which they recover, with the kinetics of recovery a good indicator of the mechanisms involved. In order to study the recovery of cells after exposure to H_2_O_2_, cells were treated with 2 mM H_2_O_2_ for 30 minutes, washed and re-suspended in fresh medium and the redox state and pH measured every two minutes. The 2 mM H_2_O_2_ dose was chosen since this concentration led to oxidation of half of the roGFP2 probe as determined in previous H_2_O_2_ experiments and led to a significant shift in *E*_GSH_ without causing cell death (Figure 4A–C). In response to H_2_O_2_ treatment (2 mM; 30 min), the cytosol, mitochondrial matrix and peroxisome displayed distinct kinetics of response and recovery (Figure 5A–C). All compartments became more oxidizing by approximately 30 to 40 mV and more acidic by 1 pH unit. In the 40 minute period studied, the *E*_GSH_ and pH in the cytosol and mitochondrial matrix recovered by 80–90%, with the cytosol recovering most rapidly, within four minutes of cells being re-suspended in fresh medium. The mitochondrial matrix exhibited slightly slower kinetics of recovery, with changes in *E*_GSH_ and pH observed 10 minutes after transfer to fresh medium. However, the peroxisome was the most severely affected compartment in terms of redox state and pH recovery. Following an initial recovery after 10 minutes in fresh medium the *E*_GSH_ in the peroxisome reached a plateau, with the *E*_GSH_ recovering only ∼25% of the change after hydrogen peroxide exposure. In the same way, in the time period studied, the pH of the peroxisome was only able to recover by 0.2 pH units and did not return to that of untreated cells. These results indicate that there are specific and individual responses in different organelles to hydrogen peroxide. Importantly, the data highlight that there is distinct spatiotemporal regulation of redox state.

In order to put these redox changes in the context of the effects of the 2 mM H_2_O_2_ treatment on the survival and growth of the cultures, cell survival in terms of ability of cells to replicate after treatment was assessed by plate counts before and after treatment, and the effects on population growth was determined by continuous monitoring of the growth of untreated and treated cultures. After treatment with 2 mM H_2_O_2_ for 30 minutes 77% of the wild-type cells survived to be able to produce a colony. The growth curves for the treated and untreated cells are given in Figure S1A. The untreated culture showed no lag phase, while the population of treated cells had a lag of 139 minutes (uncorrected for cells that are not going to replicate). The doubling time was 130 minutes, hence the correction for 77% survival would be 48 minutes for the cells to grow to the starting OD. The lag phase for the overall population after 2 mM treatment was therefore about 90 minutes. This indicates that the population of H_2_O_2_-treated cells takes about 90 minutes to recover full growth rate, which is longer than the time it takes for the population to recover the cytoplasmic and mitochondrial redox potentials. Some cells may recover earlier given the sigmoidal nature of the growth curve, although there was little increase in growth of the treated culture in the first 45 minutes after the treatment. These data indicate that cells recover their cytoplasmic and mitochondrial *E*_GSH_ before they resume growth at a normal rate, but this need not imply a causal relationship between redox recovery and delay in growth.

### Compartmental Redox State in Response to Heat Induced Stress

**Figure 6:**
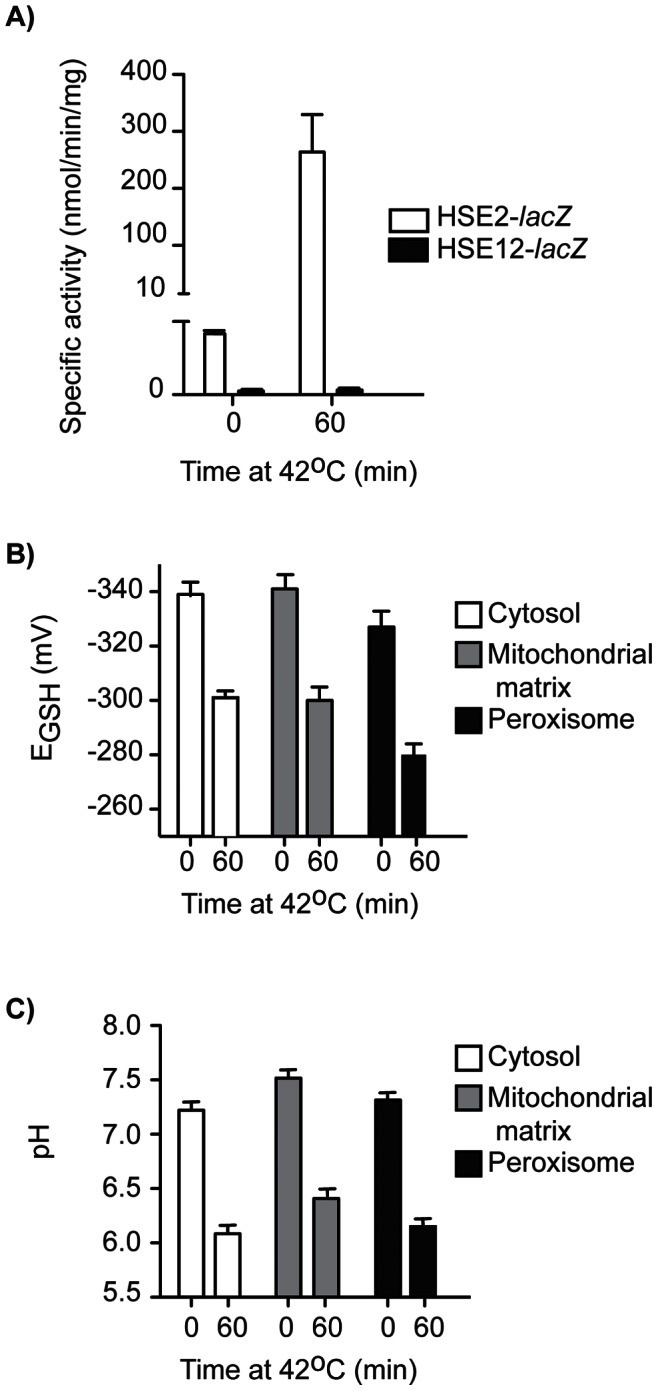
Effect of heat shock on compartmental *E*_GSH_ and pH. A) Wild-type cells with the HSE2-lacZ (heat-shock response induction reporter) construct were pregrown in SC_URA_ (48 h; 30°C; 600 rpm) and then inoculated in SC_URA_ (A_600_ = 0.001) and grown (25°C; 600 rpm) until exponential phase (A_600_ = ∼0.5). Cells were then shifted to 42°C for 60 min and harvested. β-galactosidase and total protein (Bradford) assays were carried out on cell pellets and specific activity determined. B-C) Cells were pregrown in SC_URA_ (48 h; 30°C; 600 rpm), inoculated in SC_URA_ (A_600_ = 0.001) and grown (25°C; 600 rpm) until exponential phase (A_600_ = ∼0.5). Cells were then shifted to 42°C for 60 min and *E*_GSH_ B) and pH C) analyzed via flow cytometry. 10,000 cells were counted for each condition. Each experiment was conducted in triplicate with error bars representing the standard deviation of three experiments.

Many chemical and environmental stress conditions trigger comparable transcriptional responses and the common component of these has been termed the environmental stress response (ESR) [9]. An important feature of the environmental stress response is up-regulation of systems for the detoxification of reactive oxygen species indicating that oxidative stress may be an important component in the response of cells to a wide variety of stress conditions. For heat stress in particular this view has led to the theory that oxidative stress is a component of the response to heat-stress [36], [37]. To investigate the effect of heat shock on compartmental redox state and pH, cells separately transformed with the roGFP2 and pHluorin constructs were grown to exponential phase and subjected to thermal shock by shifting them from 25°C to 42°C for 60 min. Redox state and pH were then measured and *E*_GSH_ calculated. This heat shock regime led to a 275-fold induction of the heat shock response as measured using a heat-shock element *lacZ* reporter HSE2-lacZ [24] (Figure 6A). The HSE2-lacZ construct is an artificial heat-shock promoter coupled to the *lacZ* gene, which is induced under conditions of heat stress leading to production of ß-galactosidase. Increased ß-galactosidase activity correlates with increased conditions of cellular heat stress.

*E*_GSH_ in all compartments tested became significantly more oxidized by the temperature shift from 25°C to 42°C (*p*<0.01), with a 40 mV decrease in *E*_GSH_ observed after 60 minutes at 42°C in all compartments (Figure 6B). Interestingly, intracellular pH was significantly affected by heat shock (Figure 6B). Heat shock resulted in every compartment tested becoming significantly more acidic (p<0.01) and this shift in intracellular pH modulated the large change in *E*_GSH_ rather than direct changes to the glutathione couple since the roGFP2 R_405/488_ ratio did not undergo a major change. A recent screen (Jarolim S and Dawes IW, unpublished data) has also identified numerous genes required for vacuolar H^+^-ATPase activity to be essential for survival to heat stress. Since the H^+^-ATPase functions to pump protons from the cell, this highlights the importance of cellular functions restoring pH homeostasis as central in the cellular response to heat stress. Significantly, these data highlight a stress condition that can mediate changes in redox state, primarily through changes in intracellular pH rather than through directly disturbance of a redox couple(s).

### Compartmental Redox State in Response to Sorbitol, Sodium and Potassium Induced Stress

To determine if other stresses affected redox homeostasis, we analyzed the redox state of the cytosol, mitochondrial matrix and peroxisome in wild-type cells after exposure to hyperosmotic or salt-induced stress. Wild-type cells transformed with roGFP2 or pHluorin constructs were grown to exponential phase and treated with 1.8 M sorbitol, or 0.9 M NaCl or 1.3 M KCl for 60 minutes. Redox state and pH were then measured and *E*_GSH_ calculated. All concentrations and times chosen have been reported to induce known osmotic shock-responsive pathways and indicators of stress such as P-bodies [10], [38]. Treatment with sorbitol, NaCl and KCl did not significantly affect the redox state or pH of the cytosol, mitochondrial matrix or peroxisome (Table 2). Results indicate that while sorbitol-, sodium-, and potassium-induced stress may lead to the up-regulation of some oxidative stress-response genes [9], [10], [38], [39], these stresses do not directly affect the sub-cellular redox state measured using roGFP2.

### Redox State and Hydrogen Peroxide-mediated Adaptation

Cells pre-treated with a low sub-lethal level of stress have an increased ability to survive subsequent challenge [31], [40]. Cellular adaptation has been observed in response to numerous ROS or ROS-generating agents such as H_2_O_2_ [31], menadione [40], [41] and linoleic acid hydroperoxide [42]. While the effect of adaptation to H_2_O_2_ on survival has been well studied [43], the effect of the adaptation process on organelles and redox state is unknown. To investigate whether redox changes may be involved in the adaptive process, wild-type cells expressing roGFP2 or pHluorin were treated with a low adaptive dose of H_2_O_2_ (0.2 mM) for one hour and subsequently challenged with 2 mM H_2_O_2_ for 30 minutes. Redox state and pH were then calculated (Table 3). As in previous experiments, 2 mM H_2_O_2_ was chosen since it significantly affected *E*_GSH_ in all compartments tested and this dose has been used previously to examine how adaptation affects survivability [31]. 0.2 mM hydrogen peroxide concentration was chosen as an adaptive dose using the adaptation data from Ng et al [43]. Additionally, cells treated with 0.2 mM hydrogen peroxide did not show a shift in *E*_GSH_ during 60 minutes treatment. As controls, cells were treated with each H_2_O_2_ concentration (0.2 mM or 2 mM) individually to ascertain the effects of each on redox state and pH. Only a slight change in E_GSH_ was observed in cells treated with the adaptive dose of H_2_O_2_ (0.2 mM), indicating that changes in *E*_GSH_ are not likely to be the trigger for an adaptive response. Treatment with 2 mM H_2_O_2_ led to a large and significant decrease in *E*_GSH_ (70 mV). However, *E*_GSH_ in all compartments remained approximately ∼10 mV higher in cells pretreated with 0.2 mM H_2_O_2_ and subsequently challenged with 2 mM H_2_O_2_ as opposed to cells that were only exposed to 2 mM H_2_O_2_. This difference in *E*_GSH_ (10–12 mV) in each compartment) was statistically significant, and hence *E*_GSH_ might be involved in the later stages of an adaptive response.

**Table 3:** *E*_GSH_ and pH of wild type, *yap1* and *skn7* cells grown in SC_URA_ to exponential phase and during the adaptive response to hydrogen peroxide treatment.

	*E*_GSH_ ± SD (−mV)			pH ± SD		
	Cytosol	Matrix	Peroxisome	Cytosol	Matrix	Peroxisome
**Wild type**						
Control	350±3	357±3	346±4	7.5±0.2	7.7±0.1	8.0±0.2
Adaptive dose(0.2 mM; 60 min)	348±3	354±4	340±4	7.5±0.1	7.7±0.2	8.0±0.2
Lethal dose(2 mM; 30 min)	280±5	285±3	278±3	6.0±0.1	6.1±0.1	6.0±0.1
Adaptive+lethal dose	292±4	296±4	289±3	6.0±0.1	6.1±0.1	6.1±0.2
***yap1***						
Control	320±4	337±4	336±4	7.5±0.1	7.6±0.2	7.9±0.1
Adaptive dose(0.2 mM; 60 min)	318±3	334±3	335±4	7.4±0.2	7.6±0.1	8.0±0.2
Lethal dose(2 mM; 30 min)	265±5	280±4	270±3	6.0±0.2	6.1±0.2	6.0±0.1
Adaptive+lethal dose	277±4	289±5	281±3	6.1±0.1	6.1±0.2	6.0±0.1
***skn7***						
Control	319±5	340±6	337±5	7.5±0.1	7.7±0.1	8.0±0.2
Adaptive dose(0.2 mM; 60 min)	321±4	339±5	333±4	7.5±0.1	7.7±0.2	8.0±0.1
Lethal dose(2 mM; 30 min)	281±3	283±2	279±5	6.1±0.1	6.0±0.2	6.0±0.1
Adaptive+lethal dose	291±5	294±3	291±4	6.0±0.2	6.1±0.1	6.0±0.2

Both the Yap1p and Skn7p transcription factors are key mediators of resistance to H_2_O_2_ and the *YAP1* and *SKN7* genes are required for a maximal adaptive response [43]. Additionally, both *YAP1* and *SKN7* have been identified as essential for the maintenance of cytosolic, mitochondrial matrix and peroxisomal redox state, indicating that both transcription factors sit at the boundary of maintenance-response systems [18]. From these data it has been concluded that expression of some genes regulated by Yap1p and Skn7p are important for steady-state redox homeostasis, while induction and activation of target genes appears important for mounting appropriate responses to oxidative stress [18]. If the redox state changes observed in the wild type after adaption with a low adaptive hydrogen peroxide dose are part of the adaptive process once the transcription factors are activated, the redox changes would not be observed in the *yap1* or *skn7* mutants. Hence, *yap1* and *skn7* mutants were treated as per the wild type and redox state and pH in the cytosol, mitochondrial matrix and peroxisome were determined (Table 3).

The data in Table 3 indicate that the *yap1* and *skn7* mutants both had significantly higher cytosolic *E*_GSH_ than the wild type (by 30 mV) and mitochondrial matrix *E*_GSH_ (by 20 mV), but there was much less difference in the peroxisomal *E*_GSH_. Previous studies with whole cells have shown that deletion of *YAP1* or *SKN7* does affect the redox state of the glutathione couple [27], [43], the present data indicate that redox homeostasis is affected by these deletions to a greater degree in the cytosol than the mitochondrial matrix and is little affected in the peroxisome for which there may be different systems responsible for maintenance of redox status.

There was very little change slight in redox state in all compartments tested in both the *yap1* and *skn7* mutants after treatment with 0.2 mM H_2_O_2_ as was found for the wild type (Table 3). Treatment with 2 mM H_2_O_2_ resulted in a decrease in cytoplasmic *E*_GSH_ by 70 mV for the wild type (to −280 mV), 55 mV (to −265 mV) for *yap1* cells and 40 mV (to −279 mV) in *skn7* cells. There were similar changes in the other two compartments, and interestingly the *E*_GSH_ in each compartment in treated cells of the wild type and mutants was around −280 mV (with the exception of the cytoplasm in the *yap1* mutant. This indicates that H_2_O_2_ treatment affects all compartments in the cell to almost the same extent regardless of whether the cell was initially in a more oxidised state due to the *yap1* or *skn7* mutation. This may reflect the extent of oxidation of glutathione by the oxidant.

In parallel with the wild type, *E*_GSH_ in all compartments remained approximately ∼10 mV higher in the *yap1* and *skn7* cells pretreated with 0.2 mM H_2_O_2_ and subsequently challenged with 2 mM H_2_O_2_ compared to cells that were only treated with 2 mM H_2_O_2_. Given that Yap1p and Skn7p have been shown to be key mediators in the adaptive process [43], and the responses of cells observed were comparable to those in the wild type, it is unlikely that redox state changes *per se* are important subsequent events in the cellular adaptation to H_2_O_2_. As an additional control, wild type, *yap1* and *skn7* cells were stained with propidium iodide directly after each hydrogen peroxide treatment to assess cell vitality and to assess the level of dead cells in the cell populations that were assessed by FACS. All cells stained had very low levels of PI staining following any hydrogen peroxide treatment indicating that there was a low level of dead cells analysed in the roGFP2 and pH analyses (Figure S2). These analyses were performed by microscopy and flow cytometry with identical results. There were, however, differences in the colony-forming ability of the wild-type (77%), *yap1* (63%) and *skn7* (35%) cells after treatment with 2 mM H_2_O_2_, indicating that while the cells maintained membrane integrity after treatment there were differences in their longer term survival. Furthermore, directly after each hydrogen peroxide treatment, wild-type, *yap1* and *skn7* cells were diluted to OD_600_ 0.05 and growth was measured continuously to establish if any of the treatments affected cell growth. All strains showed a lag in growth following treatment with 2 mM H_2_O_2_ or the combined adaptive 0.2 mM followed by 2 mM treatment, as discussed above for the wild type, and these lags were comparable between the wild type and mutants (Figure S1B–D).

## Discussion

A key limitation in redox biology has been the ability to measure the status of key redox couples at the sub-cellular level. The advent of genetically encoded fluorescent probes such as roGFP2 and pHluorin has allowed compartmental redox dynamics to be studied in an in-depth manner for the first time. While redox and pH sensors have been targeted to various organelles [14], [30], [44], [45], this is the first study to utilise a redox and pH sensor in tandem to specifically monitor the sub-cellular GSSG/2GSH redox couple during various physiological states and stress conditions. By using roGFP2 and pHluorin localized to the cytosol, mitochondrial matrix and peroxisome, a better understanding can be gained of the precise nature of sub-cellular redox homeostasis during changing cellular settings especially during stress conditions.

The *E*_GSH_ values obtained for the cytosol, mitochondrial matrix and peroxisome in this study are more reducing than previously calculated [27]. Using a whole-cell method, Drakulic and colleagues estimated the *E*_GSH_ of wild-type cells to be approximately −230 mV, which is ∼100 mV more positive than the *E*_GSH_ values reported here. The difference can be explained by analyses provided by roGFP2 and pHluorin being compartment-specific rather than ‘whole-cell’ as in previous studies and that an assumed cellular pH of 7 was used previously when calculating redox state. Use of another redox-sensitive fluorescent probe rxYFP has estimated cytosolic *E*_GSH_ to be −274 mV [29] and −286 mV [2] and mitochondrial matrix *E*_GSH_ to be - 296 mV [2] in wild-type cells. While these values are significantly more reducing than the previous whole-cell estimates, they are still considerably different to the values obtained in this study since compartmental pH was not measured. In the present study the pH in both the cytosol and mitochondrial matrix was found to be slightly more basic by 0.2–0.3 pH units than previously estimated in *S. cerevisiae* using less specific, whole-cell methods. Previous values for the pH of the cytosol and mitochondrial matrix have ranged from pH 7–7.2 [23], [30] and pH 7.2–7.5 [30] respectively. The outcome of a more basic sub-cellular pH is significant, leading to a more reducing redox environment based on the GSSG/2GSH couple.

The standard half-cell reduction potential of the glutathione couple is −240 mV [8], however a wild-type *E*_GSH_ value of approximately −340 mV was obtained in the three compartments tested. This puts the cellular redox state closer to that of the NADP^+^/NADPH and NAD^+^/NADH couples which have half-cell reduction potentials of −315 mV [46], [47] and −316 mV [8], [47] respectively. It may appear unfavourable for NADPH to reduce GSSG to GSH since the NADP^+^/NADPH couple has an E_0_′ value of −315 mV while the *E*_GSH_ of the cellular compartments tested here was approximately −340 mV. However the concentration of redox species must be taken into account when determining the intracellular half-cell redox potential of any redox couple [8]. Using an NADP^+^/NADPH ratio of 100∶1 [48] the *E*_NADPH_ is −374 mV. The highly reducing nature of the cytosol, mitochondrial matrix and peroxisome also reflects the critical role of NADPH in providing the primary source of reducing equivalents for the glutathione couple, with the need to maintain a high NADPH:NADP^+^ ratio driving the glutathione couple and to maintain redox buffering.

A central question in redox biology is whether compartments have distinct redox regulation and the possible crosstalk that may exist between organelles in the overall maintenance of cellular redox homeostasis. Despite all organelles tested being highly reducing and slightly alkaline, there were differences in steady state *E*_GSH_ and pH between all compartments. Following H_2_O_2_ challenge sub-cellular compartments respond distinctly - the cytosol, mitochondrial matrix and peroxisome all responded and recovered differently after treatment with 2 mM H_2_O_2_ for 20 min. It is highly probable that the distinct nature of the response and recovery of sub-cellular redox environments to redox state change is a reflection of the redox buffering and antioxidant capabilities of each compartment. The peroxisome in *S. cerevisiae* is known to only contain three antioxidant-related enzymes, a catalase, a NADP^+^-dependent isocitrate dehydrogenase and an omega-class glutathione transferase encoded by *CTA1* [49]–[51], *IDP3* [52] and *GTO1* [51] respectively. The lack of multiple systems for restoring redox state may explain the failure of peroxisomes to re-equilibrate the GSSG/2GSH couple in the time studied. This highlights the importance of studying sub-cellular compartments individually rather than viewing the cell as having a singular redox environment.

Overall these data favour the idea that changes in antioxidant enzymes or redox–related genes observed through transcriptomic or phenotypic analyses should not be used to extrapolate to changes to cellular or sub-cellular redox state. Out of all the stresses tested in this study, only two (H_2_O_2_ and heat stress) were found to alter sub-cellular redox state despite all stresses being previously associated with redox dysfunction. Interestingly, H_2_O_2_ and heat stress modulated changes to *E*_GSH_ in different ways. Under H_2_O_2_ stress both the GSSG/2GSH couple and pH changed, contributing to the overall change in *E*_GSH_. Conversely, after heat stress there was little change observed in the GSSG/2GSH couple despite reports of decreased thermotolerance in cells mutated in key antioxidant enzymes [37], [53]. The changes to intracellular pH observed were large enough to alter the intracellular redox state significantly in the cytosol, mitochondrial matrix and peroxisome. These data highlight the role of pH in redox buffering systems, and pH homeostasis in cells is an important component to study when attempting to identify mechanisms affecting redox state changes. It is possible that there are stresses that have not been conventionally linked with redox dysfunction that do perturb redox state through intracellular pH changes.

The data also raises some interesting questions about how intracellular redox state is viewed. In cells there exist multiple redox couples that are important and relatively independent thermodynamically, with varying kinetic interactions including the thioredoxin couple and protein thiols. While roGFP2 provides an excellent read-out of the GSSG/2GSH couple, the stresses tested may affect other redox couples significantly. For example, paraquat generates the superoxide anion radical that is deleterious to the cell, yet paraquat-treated cells did not exhibit a change in the glutathione couple measured by roGFP2. It is likely that paraquat treatment and subsequent superoxide anion radical formation affect cellular redox homeostasis in some manner, but the data presented here indicated that is unlikely that this directly involves the GSSG/2GSH couple. Examples such as these highlight the need for specificity when discussing the redox environment and redox dysfunction. The stresses tested in this study were stress conditions that have been conventionally associated with redox state changes. However, glutathione homeostasis is also important in iron metabolism [17]; [54], iron-sulfur transport and in the detoxification of reactive electrophiles such as heavy metals and xenobiotics. Furthermore, processes that affect iron metabolism (such as iron starvation) or the level of reactive electrophiles present in cells may in turn alter compartmental glutathione concentrations conditions. It would be of interest to test these stresses to understand their effect on compartmental redox state based on the glutathione couple.

There have been many studies investigating the molecular mechanisms underpinning the ability of cells to adapt to various stress conditions. In the study of Ng et al (2008) [43], *yap1* and *skn7* mutants were identified as having a much reduced but still detectable ability to adapt to H_2_O_2_. Given the importance of Yap1p and Skn7p in redox homeostasis, it was hypothesized that a component of the adaptive response is dependent on the expression of genes important for redox regulation, with a resetting of redox homeostasis required for adaptation. However, the results of this current study indicate that during adaption to H_2_O_2_, redox homeostasis can occur independently of the Yap1/Skn7 transcription factors. The resistance to changes in *E*_GSH_ brought about by 0.2 mM H_2_O_2_ pre-treatment may reflect the basal level of adaptive ability that *yap1* and *skn7* cells can undergo but this level is not enough to change the level of survival after H_2_O_2_ treatment.

The parallel analyses of cytosolic, mitochondrial matrix and peroxisomal redox states using roGFP2 and pHluorin carried out in this study have allowed for the monitoring of distinct glutathione redox changes under various conditions. It is clear that changes in sub-cellular redox state occur in a distinct manner in response to stress, with each organelle a discrete redox unit. Data from Dardalhon et al [34] also exemplifies this, since they show that the cytosolic and nuclear redox states are to some extent regulated independently, with both the thioredoxin and glutathione systems important. There is also some conflicting data indicating that the redox state of the mitochondrial inter-membrane space (IMS) may be regulated independently from the cytosol and mitochondrial matrix [2], [17], [55] - the variation in reported IMS *E*_GSH_ values may possibly be due to strain background differences. The data presented in this study reinforce that sub-cellular redox state can be affected through alterations to the glutathione couple, pH or both. In particular the data reinforces the idea that changes to indicators of oxidative stress such as antioxidant enzymes do not always accurately reflect changes in the sub-cellular redox environment and challenges current ideas around the role of cellular redox status in stress responses.

## Supporting Information

Figure S1 **Cell growth of WT,** ***yap1*** **and** ***skn7*** **cells after various hydrogen peroxide treatments:** Cells were pregrown in SC_URA_ (48 h; 30°C; 600 rpm) inoculated in SC_URA_ (A_600_ = 0.001) and grown (25°C; 600 rpm) until exponential phase (A_600_ = ∼0.5). Cells were left untreated or treated with hydrogen peroxide (0.2 mM for 60 min OR 2 mM for 30 min; OR 0.2 mM for 60 min followed by 2 mM for 30 min; 25°C). After hydrogen peroxide treatment, cells were diluted to OD_600_ = 0.05 and OD_600_ was measured every 15 min using a Bioscreen C.
(TIF)

Figure S2 **Propidium iodide staining of WT,** ***yap1*** **and** ***skn7*** **cells after various hydrogen peroxide treatments:** Cells were pregrown in SC_URA_ (48 h; 30°C; 600 rpm) and then inoculated in SC_URA_ (A_600_ = 0.001) and grown (25°C; 600 rpm) until exponential phase (A_600_ = ∼0.5). Cells were harvested by centrifugation, resuspended in phosphate buffered saline (PBS), stained with propidium iodide (10 ug/ml) in the dark for 20 min. Cells were washed twice with PBS and level of PI staining analysed by microscopy and flow cytometry. Cells were left untreated or treated with hydrogen peroxide (0.2 mM for 60 min OR 2 mM for 30 min; OR 0.2 mM for 60 min followed by 2 mM for 30 min; 25°C). After hydrogen peroxide treatment the level of PI staining was analysed by microscopy and flow cytometry.
(TIF)

Table S1 **Supplements for synthetic complete (SC) medium.**
(DOC)

Table S2 **Primers used in generation of mitochondrial matrix and peroxisomal targeted pHluorin probes.**
(DOCX)
