# Supplementary figures and images for: Distinct Redox Regulation in Sub-Cellular Compartments in Response to Various Stress Conditions in Saccharomyces cerevisiae
Source: PLoS One. 2013 Jun 7;8(6):e65240. doi: 10.1371/journal.pone.0065240 (PMC3676407; doi:10.1371/journal.pone.0065240)

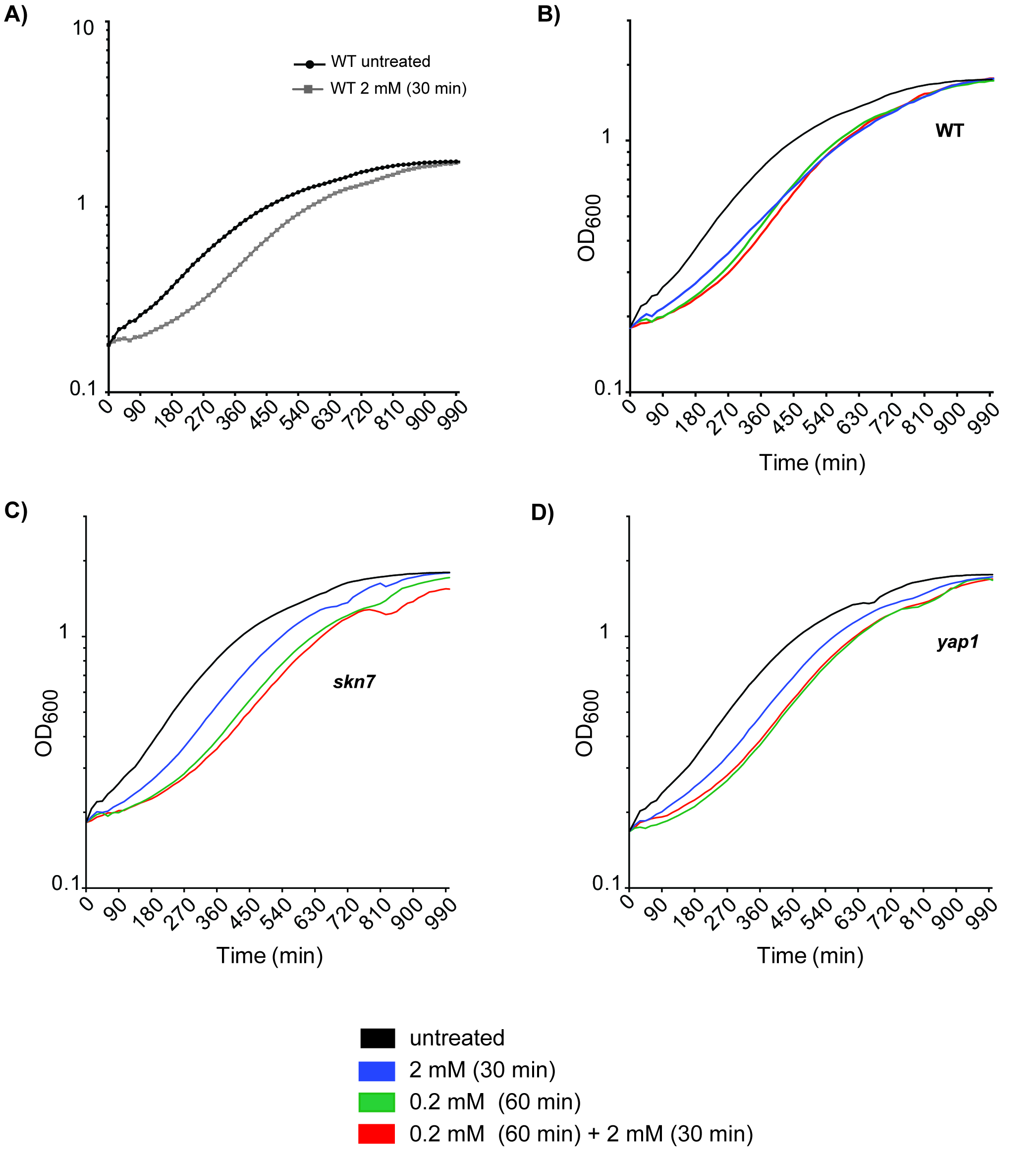

Supplement: Figure S1 — Cell growth of WT, yap1 and skn7 cells after various hydrogen peroxide treatments: Cells were pregrown in SCURA (48 h; 30°C; 600 rpm) inoculated in SCURA (A600 = 0.001) and grown (25°C; 600 rpm) until exponential phase (A600 = ∼0.5). Cells were left untreated or treated with hydrogen peroxide (0.2 mM for 60 min OR 2 mM for 30 min; OR 0.2 mM for 60 min followed by 2 mM for 30 min; 25°C). After hydrogen peroxide treatment, cells were diluted to OD600 = 0.05 and OD600 was measured every 15 min using a Bioscreen C. (TIF) [file pone.0065240.s001.tif]

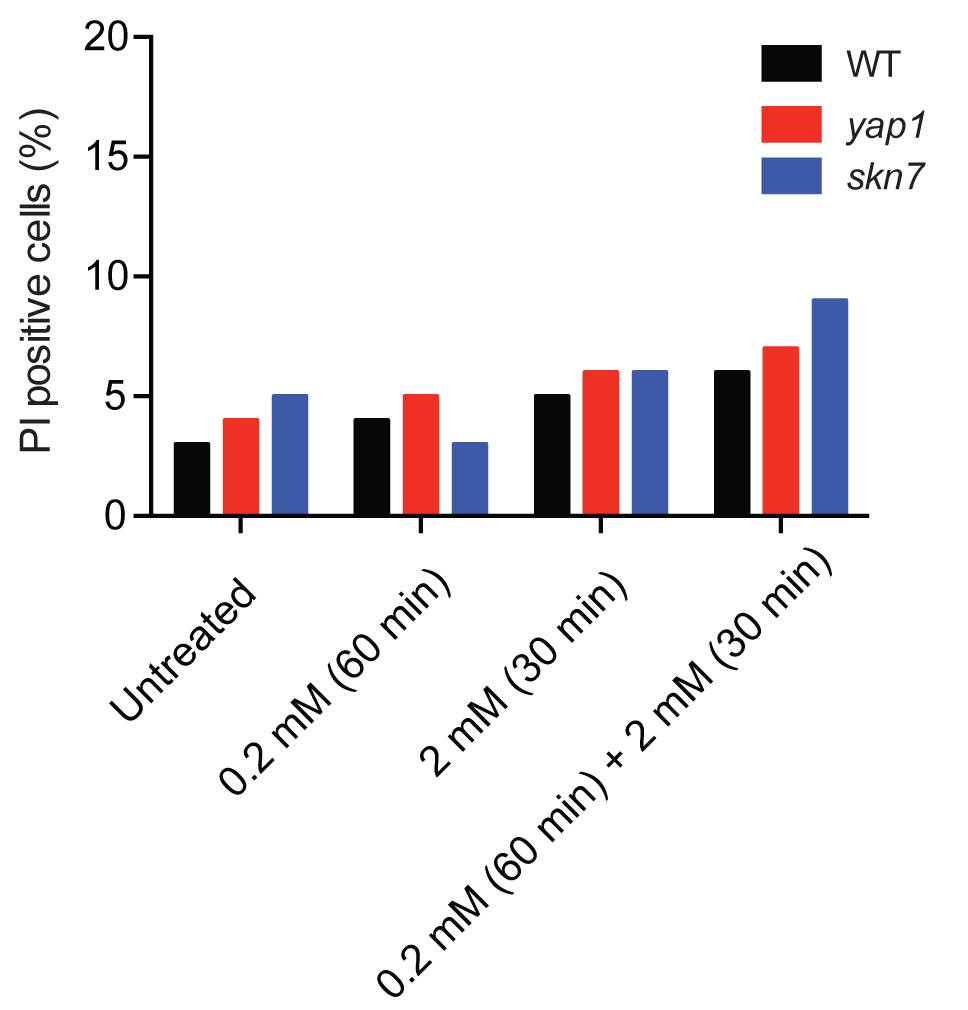

Supplement: Figure S2 — Propidium iodide staining of WT, yap1 and skn7 cells after various hydrogen peroxide treatments: Cells were pregrown in SCURA (48 h; 30°C; 600 rpm) and then inoculated in SCURA (A600 = 0.001) and grown (25°C; 600 rpm) until exponential phase (A600 = ∼0.5). Cells were harvested by centrifugation, resuspended in phosphate buffered saline (PBS), stained with propidium iodide (10 ug/ml) in the dark for 20 min. Cells were washed twice with PBS and level of PI staining analysed by microscopy and flow cytometry. Cells were left untreated or treated with hydrogen peroxide (0.2 mM for 60 min OR 2 mM for 30 min; OR 0.2 mM for 60 min followed by 2 mM for 30 min; 25°C). After hydrogen peroxide treatment the level of PI staining was analysed by microscopy and flow cytometry. (TIF) [file pone.0065240.s002.tif]
