# Supplementary material for: Distinct Redox Regulation in Sub-Cellular Compartments in Response to Various Stress Conditions in Saccharomyces cerevisiae
Source: PLoS One. 2013 Jun 7;8(6):e65240. doi: 10.1371/journal.pone.0065240 (PMC3676407; doi:10.1371/journal.pone.0065240)
Supplement: Table S1 — Supplements for synthetic complete (SC) medium. (DOC) [file pone.0065240.s003.doc]

**Supplementary Figure Legends**

**Supplementary Figure 1: Cell growth of WT, *yap1* and *skn7* cells after various hydrogen peroxide treatments:** Cells were pregrown in SC_URA_ (48 h; 30^o^C; 600 rpm) inoculated in SC_URA_ (A_600_ = 0.001) and grown (25^o^C; 600 rpm) until exponential phase (A_600_=~0.5). Cells were left untreated or treated with hydrogen peroxide (0.2 mM for 60 min OR 2 mM for 30 min; OR 0.2 mM for 60 min followed by 2 mM for 30 min; 25^o^C). After hydrogen peroxide treatment, cells were diluted to OD_600_=0.05 and OD_600_ was measured every 15 min using a Bioscreen C.

**Supplementary Figure 2: Propidium iodide staining of WT, *yap1* and *skn7* cells after various hydrogen peroxide treatments:** Cells were pregrown in SC_URA_ (48 h; 30^o^C; 600 rpm) and then inoculated in SC_URA_ (A_600_ = 0.001) and grown (25^o^C; 600 rpm) until exponential phase (A_600_=~0.5). Cells were harvested by centrifugation, resuspended in phosphate buffered saline (PBS), stained with propidium iodide (10 ug/ml) in the dark for 20 min. Cells were washed twice with PBS and level of PI staining analysed by microscopy and flow cytometry. Cells were left untreated or treated with hydrogen peroxide (0.2 mM for 60 min OR 2 mM for 30 min; OR 0.2 mM for 60 min followed by 2 mM for 30 min; 25^o^C). After hydrogen peroxide treatment the level of PI staining was analysed by microscopy and flow cytometry.

Supplementary Table S1: Supplements for synthetic complete (SC) medium

| **Supplement** | **Medium Concentration**  **(mg per l)** | **Supplement** | **Medium Concentration**  **(mg per l)** |
| --- | --- | --- | --- |
| Adenine | 18 | L- Leucine | 260 |
| L- Alanine | 76 | L-Lysine | 76 |
| L- Arginine | 76 | L- Methionine | 76 |
| L- Asparagine | 76 | L- Phenylalanine | 76 |
| L- Aspartic acid | 76 | L- Proline | 76 |
| L- Cysteine | 76 | L- Serine | 76 |
| L- Glutamic acid | 76 | L- Threonine | 76 |
| L- Glutamine | 76 | L- Tryptophan | 76 |
| Glycine | 76 | L- Tyrosine | 76 |
| L- Histidine | 211 | L-Valine | 76 |
| L- Isoleucine | 76 | Uracil | 22.5 |
