# Supplementary material for: Distinct Redox Regulation in Sub-Cellular Compartments in Response to Various Stress Conditions in Saccharomyces cerevisiae
Source: PLoS One. 2013 Jun 7;8(6):e65240. doi: 10.1371/journal.pone.0065240 (PMC3676407; doi:10.1371/journal.pone.0065240)
Supplement: Table S2 — Primers used in generation of mitochondrial matrix and peroxisomal targeted pHluorin probes. (DOCX) [file pone.0065240.s004.docx]

**Supplementary Table 2: Primers used in generation of mitochondrial matrix and peroxisomal targeted pHluorin probes**

|  | Primer # | Name | Sequence |
| --- | --- | --- | --- |
| COX4-pHluorin | Primer 1 | pHluorin-attb1 | GGGGACAAGTTTGTACAAAAAAGCAGGCTTCATGAGTAAAGGAGAAGAACTTTTCACTGGA |
|  | Primer 2 | COX4R | AAGCAGATATCTAGAGCTACACAAG |
|  | Primer 3 | COX4F-pHluorin | TCTAGATATCTGCTTATGAGTAAAGGAGAAGAACTTTTCA |
|  | Primer 4 | pHluorin-attb2 | GGGGACCACTTTGTACAAGAAAGCTGGGTCTTATTTGTATAGTTCATCCA |
| pHluorin-SKL | Primer 1 | pHluorin-attb1 | GGGGACAAGTTTGTACAAAAAAGCAGGCTTCATGAGTAAAGGAGAAGAACTTTTCACTGGA |
|  | Primer 2 | pHluorin-SKLattb2S | GGGGACCACTTTGTACAAGAAAGCTGGGTTTTACAATTTAGATTTGTATAGTTCATCCATGC |
|  |  |  |  |
|  |  |  |  |

For the pHluorin construct targeted to the mitochondrial matrix, four primers were used in a two-step PCR. Primers 1 and 2 refers to the primers used to PCR amplify the leader sequence and primers 3 and 4 refer to the primers used to PCR amplify the pHluorin sequence. Primers 1 and 4 were then subsequently used to fuse the leader sequence to the pHluorin in a final PCR reaction. Primers 1 and 4 contain the *attb1* and *attb2* (required for Invitrogen Gateway® cloning) sequences respectively.

For pHluorin targeted to the peroxisome, two primers were used with an *attb1* sequence incorporated in Primer 1 and the -SKL and *attb2* sequences incorporated into Primer 2.
